# Epigenetic Control of Regulatory T Cell Stability and Function: Implications for Translation

**DOI:** 10.3389/fimmu.2022.861607

**Published:** 2022-03-02

**Authors:** Anthony M. Joudi, Carla P. Reyes Flores, Benjamin D. Singer

**Affiliations:** ^1^ Department of Medicine, Division of Pulmonary and Critical Care Medicine, Northwestern University Feinberg School of Medicine, Chicago, IL, United States; ^2^ Canning Thoracic Institute, Northwestern University Feinberg School of Medicine, Chicago, IL, United States; ^3^ Department of Biochemistry and Molecular Genetics, Northwestern University Feinberg School of Medicine, Chicago, IL, United States; ^4^ Simpson Querrey Institute for Epigenetics, Northwestern University Feinberg School of Medicine, Chicago, IL, United States

**Keywords:** regulatory T cells, plasticity, inflammation, epigenetics, DNA methylation, therapeutics

## Abstract

FoxP3^+^ regulatory T (Treg) cells maintain immune homeostasis, promote self-tolerance, and have an emerging role in resolving acute inflammation, providing tissue protection, and repairing tissue damage. Some data suggest that FoxP3^+^ T cells are plastic, exhibiting susceptibility to losing their function in inflammatory cytokine-rich microenvironments and paradoxically contributing to inflammatory pathology. As a result, plasticity may represent a barrier to Treg cell immunotherapy. Here, we discuss controversies surrounding Treg cell plasticity and explore determinants of Treg cell stability in inflammatory microenvironments, focusing on epigenetic mechanisms that clinical protocols could leverage to enhance efficacy and limit toxicity of Treg cell-based therapeutics.

## Introduction

In health, regulatory T (Treg) cells are essential for maintaining immune homeostasis and promoting self-tolerance. These powerful immuno-modulatory cells, which comprise a subset of CD4^+^ T cells expressing CD25 (IL-2Rα) and the master transcription factor FoxP3 in humans and mice, suppress immune activation *via* inhibitory cell surface molecules (e.g., CTLA-4 and PD-1) and secretion of anti-inflammatory cytokines (e.g., IL-10 and TGF-β) to dampen pro-inflammatory effector immune cell functions (1–4). Recent data demonstrate that Treg cells also coordinate resolution of inflammation, provide tissue protection, and orchestrate repair of tissue damage, potentially rendering them useful to treat acute inflammation and tissue injury (5–19). Some animal experiments and observations in humans suggest that FoxP3^+^ T cells can lose their identity and function following exposure to inflammatory cytokines, resulting in loss of the canonical Treg cell transcriptional signature and acquisition of various helper T (Th) cell pro-inflammatory functions (20–25). Hence, the possibility of Treg cell plasticity represents a barrier to incorporating Treg cells into clinical protocols.

Treg cell development in the thymus involves the establishment of a specific epigenetic landscape, which is independent of, but complimentary to, FoxP3 expression and is required for specification of Treg cell lineage identity and function (26–30). Instability of Treg cell identity and function thus results from the loss of FoxP3 expression or changes in the epigenetic landscape. Natural Treg cells (nTreg cells) originate from the thymus with these transcriptional and epigenetic requirements established, persisting as a self-renewing population in the periphery (31, 32). While nTreg cells possess robust immunosuppressive capabilities, they comprise only 5–10% of human peripheral CD4^+^ T cells, thus requiring prolonged *ex vivo* expansion times (~2–5 weeks) to use them in therapeutic transfer protocols targeting acute inflammation. These long culture times thus limit the practicality of nTreg cells to treat acute inflammatory diseases or to promote tissue protection and repair following an acute injury. As naïve T cells are significantly more abundant than nTreg cells in peripheral blood, high numbers of induced Treg (iTreg) cells—naïve CD4^+^ T cells in which FoxP3 expression and a Treg cell phenotype have been induced by TGF-β *in vitro*—are rapidly obtainable, presenting a potential alternative to nTreg cells in clinical protocols. Data from murine studies suggest that iTreg cells can be generated within a few days (30, 33–35), possibly facilitating the use of iTreg cells in therapeutic transfer protocols targeting acute inflammation and injury. Induced Treg cells lack nTreg cell-type epigenetic patterns, particularly in DNA methylation, that drive phenotypic stability (27, 29, 30). Thus, defining exploitable epigenetic mechanisms that allow for nTreg cell-level stability in iTreg cells is of particular interest in the pursuit of using iTreg cells as immunotherapy. While minor populations of some immune and non-immune cells can express FoxP3 (36), our review focuses on FoxP3^+^ T cells.

## Treg Cells as Immunotherapy

The therapeutic goals of using Treg cells to induce self-tolerance and mitigate inflammation are to ameliorate immune dysregulation using minimal or no immunosuppressive pharmacotherapy while allowing proper immune responses to take place during the host response to pathogens (37). Pilot trials of Treg cells as cellular immunotherapy in humans have provided proof-of-concept for their use in diseases of auto-reactivity—including type 1 diabetes, graft-versus-host disease, and organ allo-transplantation—with promising results (38–44). In these studies, nTreg cells were isolated from patients for subsequent re-infusion either after storage or *ex vivo* expansion. Primary strategies of isolation involve obtaining mononuclear cells from leukopheresates, peripheral whole blood, or umbilical cord blood followed by Treg cell sorting using immuno-magnetic systems or flow cytometry cell sorting (45, 46). *Ex vivo* expansion protocols achieve large, pure, and suppressive cell populations while maintaining good manufacturing practice standards (47–50). Clinical trial protocols have infused dosages as high as 5 x 10^9^ cells, which typically take 2–5 weeks to generate. To enhance Treg cell purity during expansion, several groups have studied the effect of culture in the presence of the mTOR inhibitor rapamycin, as it selectively promotes growth of CD4^+^CD25^+^FoxP3^+^ Treg cells while concomitantly inhibiting CD4^+^CD25^–^ (non-Treg) effector T cells at low doses (50, 51).

Beyond induction of self-tolerance, emerging evidence demonstrates that Treg cells orchestrate resolution of inflammation, provide tissue protection, and coordinate tissue repair following a growing list of acute insults, including lung injury due to pneumonia, muscle injury, dermal injury, and vascular endothelial injury (5–18). The tissue-protective and -reparative properties of Treg cells appear to be the result of specific inflammatory signals, such as the cytokine IL-18 and the alarmin IL-33. Growth factor receptor ligands such as amphiregulin and keratinocyte growth factor may, in part, mediate these tissue-protective and -reparative functions, which are distinct from canonical T cell receptor (TCR) stimulation-dependent Treg cell suppressive functions. Promising data support broadening the use of Treg cells for the treatment of acute inflammation and tissue injury (19). Nevertheless, some lines of evidence suggest that Treg cells can exhibit plasticity in inflamed and damaged microenvironments, resulting in loss of their identity and the potential to gain pro-inflammatory effector functions (22). Discussed below, manipulating epigenetic determinants of Treg cell stability could aid efforts to maintain their beneficial functions in inflamed and damaged tissue microenvironments while limiting the potential for conversion into pathogenic T cells.

## Epigenetic Determinants of Treg Cell Development and Stability

Epigenetic mechanisms include a set of processes that modify transcriptional patterns without altering the underlying DNA sequence, allowing for heritable changes in gene expression. DNA methylation is a dynamic epigenetic modification mediated by a family of DNA methyltransferases (DNMTs) that add methyl groups to the 5’ carbon of cytosine bases to create 5-methylcytosine (5mC), which is associated with chromatin inaccessibility and transcriptional repression (52, 53). The DNMT family member DNMT1 catalyzes maintenance DNA methylation, and ubiquitin-like containing PHD and RING finger domains 1 (UHRF1) recruits DNMT1 to hemi-methylated DNA during DNA replication, serving to maintain DNA methylation patterning in mitotic cells. DNA demethylation occurs either passively during DNA replication or *via* the catalytic activity of the ten-eleven translocation (TET) family of dioxygenases, which oxidize 5mC to 5-hydroxymethylcytosine (5hmC) and other intermediates that ultimately restore unmethylated cytosine at a given position (54). Histone modifications represent another form of dynamic epigenetic alteration to chromatin, which, in combination with the non-catalytic domains of histone-modifying proteins, modulates transcriptional activity (55). For example, enzymes that promote monomethylation of lysine 4 on histone H3 (H3K4me1) and acetylation of lysine 27 on histone H3 (H3K27ac) mark active enhancer elements and promote transcription. Importantly, cellular metabolism provides substrates for epigenetic writers and erasers (e.g., methyltransferases and demethylases) in Treg cells (56). For example, our group determined that the mitochondrial electron transport chain in Treg cells is required to prevent the accumulation of toxic metabolites such as 2-hydroxyglutarate, which inhibits α-ketoglutarate-dependent enzymes such as the TETs (57). We found that loss of mitochondrial electron transport chain complex III in Treg cells results in increased levels of 2-hydroxyglutarate, altered DNA methylation patterning, and impaired Treg cell suppressive function.

**Figure 1:**
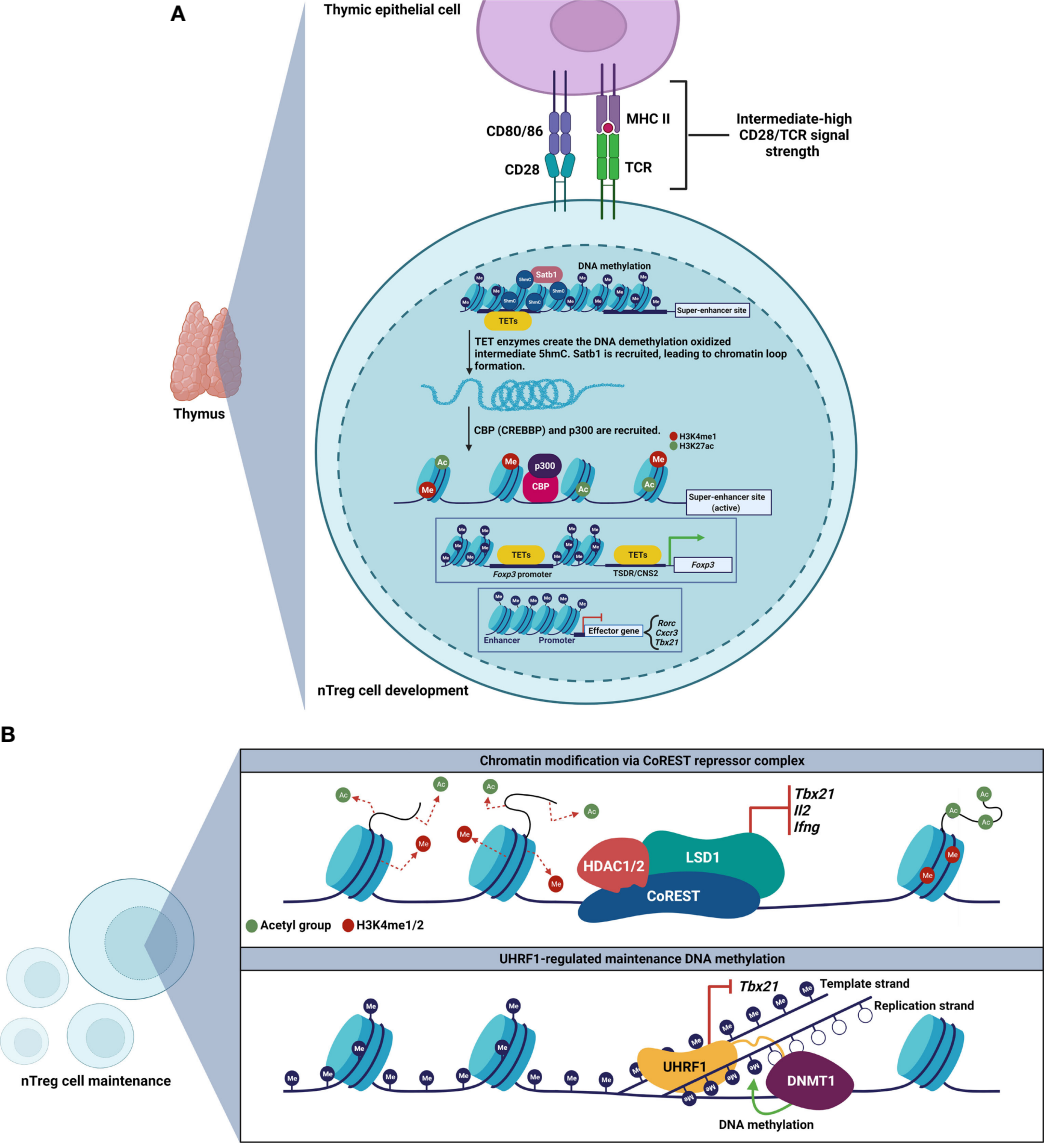
Development and maintenance of Treg cell epigenetic landscapes. **(A)** Thymic Treg cell development involves establishment of a Treg cell-specific super-enhancer landscape at *Foxp3* and other key loci. The chromatin organizer Satb1 establishes a super-enhancer landscape in Treg cells, characterized by active enhancer histone marks, and TET-mediated DNA hypomethylation. Loci encoding effector T cell signature genes are hypermethylated. **(B)** Maintenance of Treg cell epigenetic patterning requires the CoREST repressor complex (top) and the epigenetic regulator UHRF1 (bottom) to repress loci encoding inflammatory genes.

The field has now recognized that stable Treg cell phenotype and function depend on a specific epigenetic landscape to maintain lineage-defining Treg cell gene expression, including at the locus encoding FoxP3 (**Table 1**) (26–30). Accordingly, nTreg cells can be distinguished from conventional T cells and iTreg cells by characteristic DNA hypomethylation at the *Foxp3* promoter and additional elements within *Foxp3*-associated enhancer regions, such as the Treg cell-specific demethylated region (TSDR), also known as conserved noncoding DNA sequence 2 (CNS2). How the Treg cell lineage establishes and stabilizes its epigenetic signature remains an active area of investigation (**Figure 1A**). During development in the thymus, TET enzymes and HATs, such as CBP (also known as CREBBP) and p300, are recruited to modify the *Foxp3* locus for induction and maintenance of FoxP3 expression, which is followed by establishment of a Treg cell-specific gene expression profile (60, 61). Epigenetic modification at the *Foxp3* locus involves TET-mediated 5hmC accumulation at the TSDR and other key regions (59). Importantly, in the absence of these epigenetic modifications, Treg cells can lose FoxP3 expression and gain IL-17 expression.

**Table 1:** Selected epigenetic modifiers discussed in the text and their role in Treg cell development and maintenance.

Epigenetic modifier	Mechanism	Role in Treg cells
Satb1	Chromatin organizer	Establishes Treg cell-specific super-enhancer landscape (58)
TET enzymes	DNA demethylases	Induce and maintain expression of *Foxp3* and other loci (59, 60)
CBP and p300	Histone acetyltransferases (H3K27ac)	Induce and maintain expression of *Foxp3* and other loci (61)
CoREST	Epigenetic repressor complex	Represses Th1 cell signature genes (62)
UHRF1	DNA methyltransferase adapter protein	Maintains repressive DNA methylation patterning at Th1 cell signature genes to stabilize the Treg cell lineage (30); promotes proliferative capacity in colonic Treg cells (63); may regulate iTreg cell suppressive function (64)
DNMT1	Maintenance DNA methyltransferase	Required for Treg cell suppressive function (65)
EZH2	Histone methyltransferase (H3K27me3), subunit of PRC2	Deposits repressive chromatin modifications at FoxP3-bound loci (66)

See text for abbreviations.

Beyond the *Foxp3* locus, investigators have determined that Treg cell-specific super-enhancers—genomic regions with dense clustering of highly active lineage-defining enhancer elements—regulate *Foxp3* and other Treg cell lineage-defining genes (58). In thymic pre-Treg cells, the genome organizer Satb1 binds Treg cell-specific super-enhancer sites, resulting in chromatin loop formation that allows distal regulatory elements to interact with and recruit transcription factors and epigenetic modifiers to activate and stabilize the Treg cell-defining gene regulatory network. Deletion of Satb1 in double-positive thymocytes results in impaired Treg cell-super-enhancer activation and failure to induce Treg cell signature genes, leading to fatal autoimmunity *in vivo*. These studies also revealed that DNA hypomethylation is a distinguishing feature of the Treg cell-specific super-enhancer landscape in Treg cells. Moreover, our work demonstrated that the Treg cell-specific super-enhancer epigenetic pattern shown to be causally deterministic in mice is also present in Treg cells obtained from the alveolar spaces of patients with severe pneumonia (67). Thus, the Treg cell-specific super-enhancer landscape appears to be a conserved and translationally relevant epigenetic pattern, prompting clinical trials of Treg cell infusions for patients with the acute respiratory distress syndrome due to severe SARS-CoV-2 pneumonia (16, 68, 69).

The role of maintenance of epigenetic marks in stabilizing lineage identity following the initial establishment of epigenetic patterns at Treg cell-specific super-enhancers and at other important non-coding elements remains unclear (**Figure 1B**). Experimental data suggest that some chromatin organizers necessary for lineage specification are not required for lineage stability. Indeed, deletion of Satb1 in differentiated Treg cells does not lead to any changes in Treg cell numbers or phenotype, indicating that Satb1 is dispensable for Treg cell maintenance (58). In contrast, loss of the chromatin-modifying CoREST repressor complex disrupts FoxP3-driven repression of Th1 cell signature genes encoding T-BET, IL-2, and IFN-γ. Consequently, loss of CoREST results in Treg cell production of IL-2 and IFN-γ, impaired Treg cell function, and enhanced anti-tumor immunity (62).

Maintenance DNA methylation also controls Treg cell stability following FoxP3 induction in nTreg cells. We observed that loss of an epigenetic regulator responsible for maintenance DNA methylation, UHRF1, at the thymic FoxP3^+^ stage of development in nTreg cells leads to loss of FoxP3 expression and a Scurfy-like phenotype (30). We went on to determine that Treg cell-conditional deletion of UHRF1 results in failure of nTreg cells to persist after FoxP3 induction in the thymus, generating hyperinflammatory ex-FoxP3 cells in which loss of maintenance DNA methylation derepresses Th1 cell signature genes, including *Tbx21* (encodes T-BET). Interestingly, UHRF1-deficient ex-FoxP3 cells exhibit downregulation of the TET demethylases and DNA hypermethylation at core Treg cell loci, including *Foxp3*. These observations support a mechanism in which loss of maintenance DNA methylation unleashes a secondary wave of DNA methylation at core Treg cell loci to generate hyperinflammatory, Th1-skewed, ex-FoxP3 cells. Consistent with these observations, others found that constitutive deletion of the maintenance DNA methyltransferase DNMT1, but not the *de novo* methyltransferase DNMT3A, results in diminished numbers and suppressive function of Treg cells (65). Interestingly, DNMT1-deficient Treg cells maintain Treg cell-specific DNA hypomethylation patterns at *Foxp3*, and we determined that UHRF1-deficient Treg cells possess the Treg cell-specific super-enhancer landscape. Additional evidence supports that pan-T cell-specific deficiency of UHRF1 results in defective proliferation and functional maturation of colonic Treg cells (63). Thus, nTreg cells require both a canonical hypomethylation pattern as well as maintenance methylation at loci encoding inflammatory programs to stabilize their lineage identity and function. The role of maintenance DNA methylation in stabilizing iTreg cell identity and function remains less clear. Intriguingly, while we found that UHRF1 is dispensable for induction of FoxP3 expression in iTreg cells (30), others observed augmented suppressive function in iTreg cells generated from UHRF1-deficient naïve CD4^+^ T cells, even in inflammatory microenvironments (64). As inflammation may drive instability of FoxP3^+^ T cells, we will explore in the following section how microenvironmental inflammatory signals control T cell plasticity *via* their influence on epigenetic modifiers.

## Cytokine Signaling and the Epigenetics of Treg Cell Plasticity

Plasticity refers to the capacity of CD4^+^ T cells to depolarize their specialized functional programs in response to the cytokine milieu of the local microenvironment, resulting in loss of their functional identity and potential for a gained Th-skewed cell phenotype (70). Careful lineage-tracing studies in mice reported the eminent stability of the Treg cell lineage under physiologic and inflammatory conditions (31), and others have argued that the plasticity observed in FoxP3^+^ T cells in inflammatory or lymphopenic microenvironments results from cellular heterogeneity rather than reprogramming (71). Indeed, minor populations of conventional T cells can transiently express FoxP3 and then differentiate into ex-FoxP3 Th-skewed cells (72, 73). These populations retain the ability to re-express FoxP3 upon activation, a finding correlated with the demethylated status of the TSDR in the conventional T cell population and possibly the Treg cell population.

**Figure 2:**
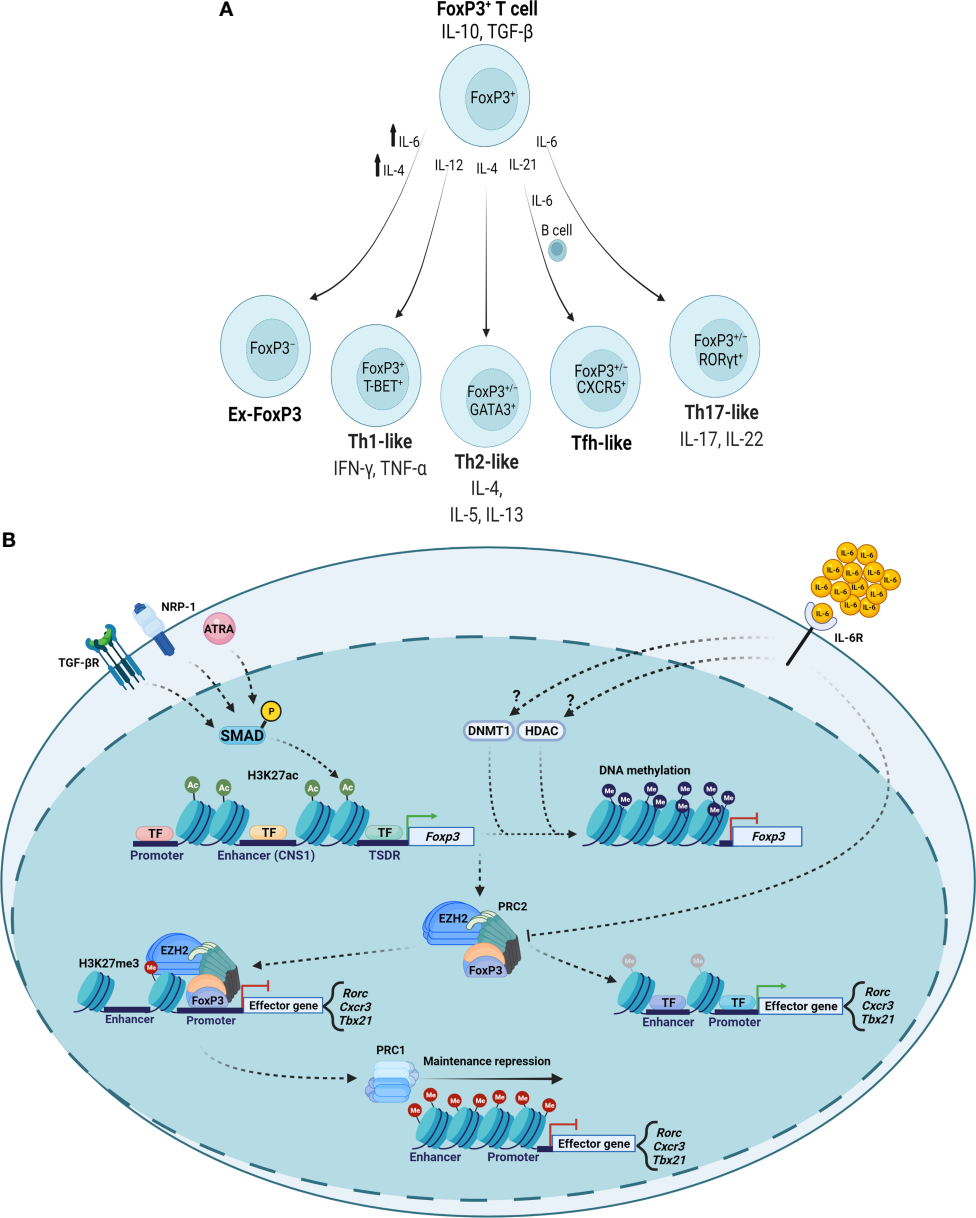
Cytokine-mediated epigenetic reprogramming of FoxP3^+^ T cell populations. **(A)** Specific cytokine microenvironments can repolarize FoxP3^+^ T cells with variable effects on FoxP3 expression and Th cell-like phenotypes. **(B)** TGF-β, NRP-1, and ATRA signal to maintain Treg cell-type epigenetic patterns. Inflammatory cytokines such as IL-6 can promote DNMT and HDAC activity to result in loss of *Foxp3* gene expression and modulate PRC complexes to depress loci encoding inflammatory genes. TF, transcription factor.

Nevertheless, several lines of evidence describe plasticity occurring in FoxP3^+^ T cells to produce ex-FoxP3 cells or FoxP3^+^ Th-like cells in response to specific signaling events (**Figure 2A**). For example, Th1-like IFN-γ-secreting FoxP3^+^ T cells exist in patients with relapsing-remitting multiple sclerosis, a finding recapitulated *in vitro* when investigators cultured Treg cells from healthy people in the presence of IL-12 (23). IL-4 signaling promotes the development of ex-FoxP3 Th2-like cells in the setting of chronic helminth infection (25). The presence of an IL-6-, IL-21-, and activated B cell-rich environment in the Peyer’s patches of mice results in FoxP3^+^ T cell transformation into cells with characteristics of follicular Th (Tfh) cells that are capable of promoting germinal center formation (21). Th17 cells express the orphan nuclear receptor RORγt and a characteristic cytokine signature, including IL-17. Regulation of the locus encoding IL-17 *via* reciprocal actions of STAT3 and STAT5 in part determines Th17-Treg cell plasticity, producing FoxP3^+^IL-17^+^ or FoxP3^+^RORγt^+^ cells (20, 74, 75). Clinically, the joints of patients with rheumatoid arthritis contain FoxP3^+^IL-17^+^ cells, which are also present in mice with experimental joint inflammation (24). These FoxP3^+^IL-17^+^ cells may represent a transitional cell population moving toward complete loss of FoxP3, as synovial fibroblast-derived IL-6 can cause CD4^+^FoxP3^+^ T cells to lose FoxP3 expression and differentiate into Th17 cells in mice with experimental inflammatory arthritis. Further, treatment of patients with rheumatoid arthritis using the IL-6R inhibitor tocilizumab resulted in significant symptomatic benefit along with decreases in circulating Th17 cells and increases in circulating Treg cells (76). Despite expressing RORγt, FoxP3^+^RORγt^+^ cells in the intestines of mice demonstrate transcriptional and epigenetic profiles more similar to FoxP3^+^RORγt^−^ cells than to FoxP3^−^RORγt^+^ cells, including demethylation at Treg cell-characteristic genes encoding FOXP3, CTLA-4, GITR, EOS, and HELIOS. FoxP3^+^RORγt^+^ cells retain suppressive function and are more suppressive than FoxP3^+^RORγt^−^ cells in a T cell transfer colitis model (77, 78). Collectively, these reports identify plasticity within FoxP3^+^ T cell populations that is induced and modified by specific cytokine microenvironments.

Data suggest that epigenetic alterations underlie the ability of FoxP3^+^ T cells to polarize in response to microenvironmental inflammatory signals (**Figure 2B**). For example, some experiments determined that IL-6 can promote DNMT1-mediated DNA methylation and that histone deacetylase (HDAC) activity can destabilize FoxP3^+^ T cells (79, 80). EZH2 (enhancer of zeste homolog 2) is the enzymatic subunit of polycomb repressive complex 2 (PRC2), which participates in histone methylation to result in transcriptional repression. PRC1 (polycomb repressive complex 1) maintains the repressed transcriptional state, interacting with chromatin by recognizing PRC2-established H3K27me3 marks in an equilibrium with other histone-modifying complexes and repressive DNA methylation (81, 82). In inflammatory microenvironments, FoxP3-containing complexes incorporate EZH2, which deposits repressive chromatin modifications at FoxP3-bound loci (66). Studies of intestinal inflammation in inflammatory bowel disease suggested a disrupted FoxP3-EZH2 physical interaction that investigators recapitulated by treatment with IL-6 (83). In the context of aging (84), we found that cell-autonomous age-related alterations in DNA methylation drive plasticity in FoxP3^+^ T cells in the inflamed lungs of aged but not young mice during recovery from influenza pneumonia (18, 85). Our studies in aged hosts revealed co-expression of Th1- and Th17-associated transcription factors (T-BET and RORγt) in lung FoxP3^+^ T cells 60 days following influenza virus infection along with expression of cognate cytokines (IFN-γ and IL-17). In contrast, other signaling events may stabilize Treg cell-type epigenetic patterns. Transcriptional and epigenetic analyses of human Treg cells from inflamed synovial joints compared with peripheral blood in pediatric patients revealed that Treg cells differentiate into effector Treg (eTreg) cells that are suppressive *in vitro* and display increased expression of core Treg cell genes (86). Importantly, epigenetic alterations in active enhancer marks, including H3K4me1 and H3K27ac, explained these transcriptional differences. CD103^+^ intestinal dendritic cells secrete all-*trans* retinoic acid (ATRA) and TGF-β to induce histone acetylation at the CNS1 region of the *FOXP3* locus to promote FoxP3 expression while restricting Th17 polarization (87). Experimental data suggest that neuropilin-1 (NRP-1) also reinforces the stability of Treg cells in inflammatory environments, as silencing of NRP-1 results in diminished FoxP3 expression with a correlative increase in DNA methylation at the TSDR (88). Collectively, several lines of evidence support that epigenetic mechanisms determine FoxP3^+^ T cell plasticity in inflammatory environments, prompting consideration of leveraging these mechanisms to promote functional stability in clinical Treg cell transfer protocols.

## Stabilizing Treg Cell Immunotherapeutic Function *via Ex Vivo* Epigenetic Modification

**Figure 3:**
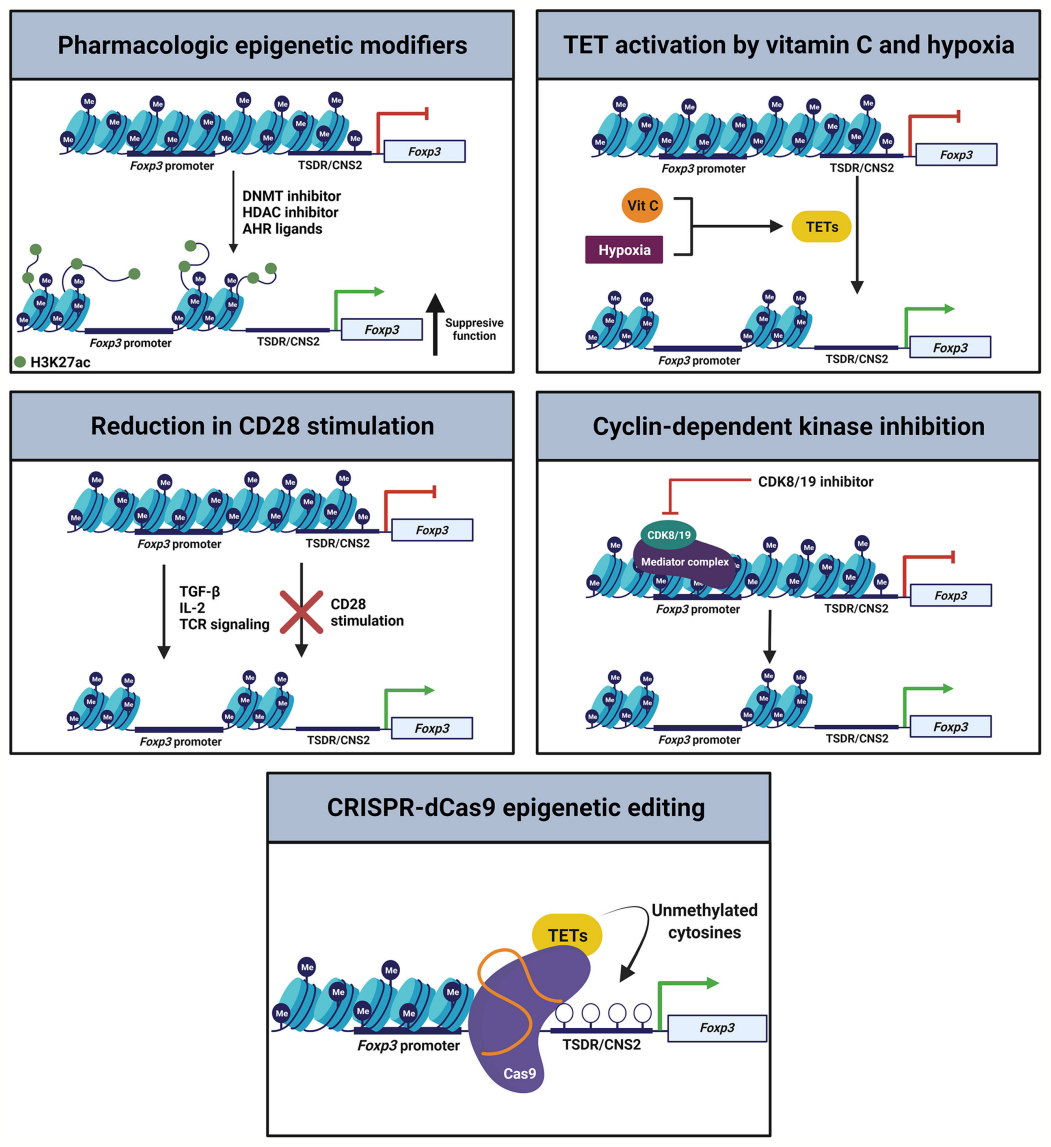
Epigenetic strategies to promote Treg cell stability. Multiple orthogonal pathways could be leveraged during *ex vivo* generation of iTreg cells or expansion of nTreg cells to promote FoxP3 expression and Treg cell stability, enhancing the efficacy of therapeutic transfer.

Treg cell plasticity may represent an adaptive feature to regulate a given microenvironment. Clinically, however, therapeutic protocols will require a greater understanding of Treg cell plasticity to maximize on-target function and limit unintended toxic inflammation. Recent mouse studies have performed *ex vivo* modifications to induced and natural Treg cells to enhance their stability in the presence of inflammatory cytokines (**Figure 3**). As inhibition of DNMTs or HDACs can induce FoxP3 expression and support Treg cell identity, DNMT or HDAC inhibitors could stabilize nTreg cells in culture before therapeutic infusion (11, 27, 79, 89–92). The DNMT adapter protein UHRF1 also remains a drug target of interest. As noted above, adoptive transfer of iTreg cells generated from UHRF1-deficient naïve CD4^+^ T cells display enhanced suppressive function in colitis models (64). In contrast, UHRF1 overexpression in T cells causes BCL6 downregulation and decreased Tfh cell differentiation, which may serve as a potential therapeutic target in systemic lupus erythematosus (93). Nevertheless, the field requires further data to determine whether modulation of UHRF1 in mature iTreg cells translates into a more stable, suppressive, and reparative state *in vivo*. Studies of HDAC inhibitors found that they are capable of promoting thymic production of nTreg cells and inducing iTreg cells *ex vivo*, leading to efficacy in attenuating inflammatory bowel disease and promoting cardiac allograft tolerance (94). Going forward, selective manipulation of HDAC subclass function may stabilize and promote nTreg and iTreg cell function in clinical trial protocols (95).

The aryl-hydrocarbon receptor (AHR) is a ligand-activated transcription factor that functions in part through interaction with epigenetic regulators, including the mediator complex (96). AHR regulates Treg-Th17 cell plasticity in mice *via* activation by specific ligands. AHR activation by 2,3,7,8-tetrachlorodibenzo-p-dioxin (TCDD) induces functional Treg cells that suppress experimental autoimmune encephalitis. Treatment of mice with TCDD also attenuates delayed hypersensitivity responses, which are associated with induction of Treg cells and suppression of Th17 cells in mesenteric lymph nodes (97). Intriguingly, treatment with AHR ligands such as TCDD or butyrate inhibits pro-inflammatory HDAC classes I and II (97). Hence, generation of iTreg cells or expansion of nTreg cells in the presence of AHR ligands may stabilize the Treg cell epigenetic landscape to maintain their identity following therapeutic transfer.

Several investigations have examined modulation of TET enzyme activity *via* treatment with the TET activator vitamin C (ascorbic acid) or culture under low oxygen conditions to enhance Treg cell induction and stability. Vitamin C facilitates demethylation of the *Foxp3* CNS2 enhancer region in a TET2/3-dependent manner to increase the stability of FoxP3 expression in TGF-β-induced Treg cells (33, 98). Further, culture of iTreg cells under low oxygen (5%) conditions facilitates CNS2 demethylation and stabilization of FoxP3 both *in vitro* and *in vivo*, a finding that correlates with increased TET expression. These post-hypoxia Treg cells exhibit stronger suppressive activity in a colitis model compared with untreated iTreg cells (34), informing potential immunotherapeutic iTreg cell induction protocols. Moreover, activation of TET enzyme activity during *ex vivo* nTreg cell expansion protocols could likewise support their stability and function.

While CD28 is essential for optimal thymic Treg cell development, CD28 is surprisingly dispensable for Treg cell induction or Treg cell-specific gene hypomethylation in the intestines of mice (35, 99). iTreg cell induction *via* TGF-β, IL-2, and TCR agonism in the absence of CD28 signaling induces nTreg cell-type DNA hypomethylation in conventional T cells while hindering skewing toward Th cell phenotypes. Data suggest that CD28 acts *via* the PKC-NF-κB signaling pathway during iTreg cell generation and that inhibition of this pathway enables *de novo* acquisition of nTreg cell-type DNA hypomethylation. Induced Treg cells generated under conditions of absent CD28 stimulation stably express FoxP3 after adoptive transfer and effectively suppress antigen-specific immune responses *in vivo* (35). Thus, potential modifications to standard nTreg cell culture practices or iTreg cell induction protocols include relatively straightforward adjustments such as reducing CD28 stimulation during cellular activation.

Cyclin-dependent kinase 8 (CDK8) and CDK19 reversibly associate with the mediator complex as well as regulate epigenetic events such as histone modification and chromatin remodeling (100, 101). Inhibition of CDK8 and CDK19 in conventional T cells induces FoxP3 expression and suppressive function independent of TGF-β signaling in antigen-stimulated effector-memory as well as naïve CD4^+^ and CD8^+^ T cells (102). Importantly, inflammatory cytokines do not appear to affect the induction of FoxP3 expression following CDK inhibition. These results suggest that CDK8 and CDK19 physiologically repress FoxP3 expression in activated conventional T cells, prompting consideration of targeting CDK8 and CDK19 in *ex vivo* iTreg cell generation or nTreg cell expansion protocols.

Finally, CRISPR-dCas9 epigenome editing systems may be of use to enhance FoxP3 stability during and following Treg cell induction or expansion. Kressler and colleagues demonstrated a transient-transfection CRISPR-dCas9-based epigenetic editing method for the selective de-methylation of the TSDR within the endogenous chromatin environment of a living cell (103). The demethylation marks were durable over weeks, including after expression of the editing complex had ceased. Consistent with prior data, however, successful FoxP3 induction was not associated with a switch to a fully functional Treg cell phenotype, highlighting importance of establishing gene expression and methylation patterns at other key loci in the Treg cell genome.

## Discussion

FoxP3^+^ Treg cells represent a powerful cell type capable of inducing self-tolerance, suppressing over-exuberant immune system activation, promoting resolution of inflammation, and effecting protection and repair of damaged tissues. Clinical trial protocols have applied Treg cell immunotherapy to disorders of auto- and allo-reactivity as well as to suppress damaging inflammation and hasten recovery from severe pneumonia. Epigenetic mechanisms, particularly those that regulate DNA methylation, control Treg cell lineage identity, stability, and function. Although the Treg cell lineage displays a strong tendency toward stability, many lines of evidence suggest that FoxP3^+^ T cells can exhibit plasticity in inflammatory microenvironments, with investigators observing both loss of canonical suppressive function and gain of inflammatory effector functions. Going forward, manipulating the epigenetic state of Treg cells *ex vivo* prior to infusion could stabilize their identity and function to enhance clinical efficacy while limiting the potential for off-target effects.

## Author Contributions

AJ and BS conceptualized the manuscript, wrote the first draft of the manuscript, edited the manuscript, and edited the figures. CR provided essential conceptual input, edited the manuscript, and conceptualized and created the figures. Conceptualization: AJ and BS. Visualization: AJ, CR, and BS. Funding acquisition: BS. Supervision: BS. Writing – original draft: AJ and BS. Writing – review & editing: AJ, CR, and BS. All authors contributed to the article and approved the submitted version.

## Funding

National Institutes of Health grant R01HL149883 (BS), National Institutes of Health grant R01HL153122 (BS), National Institutes of Health grant P01HL154998 (BS), National Institutes of Health grant P01AG049665 (BS), National Institutes of Health grant U19AI135964 (BS). The opinions expressed in this article are those of the authors and do not represent any position or policy of the National Institutes of Health.

## Conflict of Interest

BS holds United States Patent No. US 10,905,706 B2, “Compositions and Methods to Accelerate Resolution of Acute Lung Inflammation,” and serves on the Scientific Advisory Board of Zoe Biosciences, in which he holds stock options.

The remaining authors declare that the research was conducted in the absence of any commercial or financial relationships that could be construed as a potential conflict of interest.

## Publisher’s Note

All claims expressed in this article are solely those of the authors and do not necessarily represent those of their affiliated organizations, or those of the publisher, the editors and the reviewers. Any product that may be evaluated in this article, or claim that may be made by its manufacturer, is not guaranteed or endorsed by the publisher.
